# Revisiting Experimental Models of Diabetic Nephropathy

**DOI:** 10.3390/ijms21103587

**Published:** 2020-05-19

**Authors:** Anna Giralt-López, Mireia Molina-Van den Bosch, Ander Vergara, Clara García-Carro, Daniel Seron, Conxita Jacobs-Cachá, Maria José Soler

**Affiliations:** 1 Nephrology Research Group, Vall d’Hebrón Institut de Recerca, 08035 Barcelona, Spain; anna.giralt@alumni.vhir.org (A.G.-L.); mireiamoli@gmail.com (M.M.-V.d.B.); vergara.ander@gmail.com (A.V.); clara.garcia@vhebron.net (C.G.-C.); dseron@vhebron.net (D.S.); 2 Nephrology Department, Vall d’Hebrón Hospital, 08035 Barcelona, Spain

**Keywords:** diabetic nephropathy, experimental models of DN, renal function, histological lesions

## Abstract

Diabetes prevalence is constantly increasing and, nowadays, it affects more than 350 million people worldwide. Therefore, the prevalence of diabetic nephropathy (DN) has also increased, becoming the main cause of end-stage renal disease (ESRD) in the developed world. DN is characterized by albuminuria, a decline in glomerular filtration rate (GFR), hypertension, mesangial matrix expansion, glomerular basement membrane thickening, and tubulointerstitial fibrosis. The therapeutic advances in the last years have been able to modify and delay the natural course of diabetic kidney disease (DKD). Nevertheless, there is still an urgent need to characterize the pathways that are involved in DN, identify risk biomarkers and prevent kidney failure in diabetic patients. Rodent models provide valuable information regarding how DN is set and its progression through time. Despite the utility of these models, kidney disease progression depends on the diabetes induction method and susceptibility to diabetes of each experimental strain. The classical DN murine models (Streptozotocin-induced, Akita, or obese type 2 models) do not develop all of the typical DN features. For this reason, many models have been crossed to a susceptible genetic background. Knockout and transgenic strains have also been created to generate more robust models. In this review, we will focus on the description of the new DN rodent models and, additionally, we will provide an overview of the available methods for renal phenotyping.

## 1. Introduction

Diabetes is an increasing disease with more than 350 million people affected worldwide, and it has become an important public health challenge. By 2035 it is projected to reach 600 million people affected, being diabetic more than one in ten adults [1]. Type 2 diabetes is the most common form with an 85–95% incidence of all diabetes cases. The diabetes epidemic has also increased diabetic nephropathy (DN), becoming the latter an increasing health problem and the first cause of chronic kidney disease in the developed world [2]. The molecular pathogenesis of DN is far from being completely understood. Since 1970, advances in hyperglycemia, hypertension, and dyslipidemia treatment have in part change the course of diabetic kidney disease (DKD). Especially, two groups of antihypertensive drugs that block the renin-angiotensin system (RAS)—the angiotensin converting enzyme inhibitors (ACEi) and angiotensin receptor blockers (ARBs)—proved to be effective for delaying its progression. In the last five years, two groups of antidiabetic drugs—the sodium-glucose cotransporter 2 inhibitors (SGLT2i) and Glucagon-like peptide-1 receptor agonists (GLP-1 RA)—have also demonstrated promising results in terms of renal protection [3]. However, none of these treatments has demonstrated to completely stop disease progression, and cardiovascular (CV) mortality continues to grow in DKD patients. For these reasons, there is still an urgent need to find novel risk biomarkers and treatments to prevent DN.

The lack of experimental models that reproduce all structural and functional alterations of human DN is one of the barriers to solve these clinical needs in basic research. Animal models have been shown to provide some information about how DN starts and its progression through time. Therefore, they are helpful to decode, at least in part, the pathogenesis of human DN. In addition, they are useful for unraveling how new treatments work, identifying possible side effects or new pathways by which these medications act. Of note is that two of the antihypertensive drug groups that proved to delay DN progression—ACEi and ARB—have been widely studied in experimental models of DKD. The results from those studies helped to characterize different pathways that are involved in DKD, including RAS [4]. Presently, the use of SGLT2i and their combination with ACEi and ARBs are also being studied in DN models [5,6,7].

The mouse has become the main experimental model used in diabetes complications, including DN, due to their easy genetic manipulation, low cost, short gestation times, and high genetic homology with humans [8,9]. However, murine models have some limitations, as most of them just replicate early stages of diabetic kidney disease. In fact, DN classical models manifest modest albuminuria, glomerular hypertrophy, and little mesangial matrix expansion, which are features of incipient human DKD [10]. Moreover, glomerulosclerosis, tubular atrophy or interstitial fibrosis are rare in these animals. To solve this issue, in the last two decades, researchers have tried to create more severe variants of diabetic mice that develop overt DKD lesions. The aim of this review is to evaluate new rodent models of DN, identify their limitations and their main use in preclinical studies and basic research. Along with this topic, we also introduce the currently employed methods for the DN phenotyping. We discuss procedures used to assess renal function and measure renal lesions in diabetic mice.

## 2. Classical and New Models of Experimental DN

Rodents are usually chosen as animal models of diabetic kidney disease. However, most of the models show a different degree of resistance to the development of DN, as the susceptibility to nephropathy is clearly influenced by the genetic background of the strain. Nowadays, different rodent strains have been developed to achieve a suitable model that mimics human DN features [11]. In 2001, the Animal Models of Diabetic Complications Consortium (AMDCC) was created with the aim to characterize and define validation criteria of animal models with diabetes key complications, such as DN or CV disease [12]. Although a mice variant that develops all lesions of human DKD has not yet been developed, the consortium established the optimal characteristics for a DN model (Table 1) [13]. When compared with age and gender-matched controls of the same strain, the DN models should show more than a 10-fold increase in albuminuria, more than 50% decline in glomerular filtration rate over the lifetime, systemic hypertension, advanced mesangial matrix expansion with or without nodular sclerosis, and mesangiolysis, arteriolar hyalinosis, glomerular basement membrane (GBM) thickening—more than 50% over baseline—and tubulointerstitial fibrosis. Nevertheless, classical rodent models of DN, such as streptozotocin (STZ) induced, Akita, NOD, or obese type 2 models, only develop incipient lesions of DN [8].

The streptozotocin (STZ)-mice model is the most widely used model of type 1 diabetes. STZ is a compound that is especially toxic for the β-pancreatic cells and, therefore, since the early 80s it has been widely used to induce type 1 diabetes to mice and rats. However, the main problem of this diabetic model is that STZ also exerts toxicity in the renal tubular cells, thus the renal lesions might also be attributed to the direct STZ toxicity in the kidney [14,15,16]. The Akita^Ins2+^ mouse model carries an autosomal-dominant, spontaneous point mutation in the preproinsulin gene (Ins2), which causes selective toxicity to the pancreatic β-cells, leading to their dysfunction and cell death. This mouse is often regarded as an excellent type 1 model of DN, as the disease arises without any chemical intervention and it provides a better reflection of the progressive kidney disease pathology [17]. These mice develop pronounced renal-morphological changes that are similar to human DN, including hyperglycaemia, mesangial area expansion, and albuminuria. However, the severity of renal injury is highly dependent on the mouse strain [18,19]. Among the type 1 diabetic models, the non-obese diabetic mice (NOD mice) mimic the pathogenesis of human type 1 diabetes. This mouse develops autoimmune destruction of islet cells recapitulating some characteristics of type 1 diabetes mellitus in humans. The extent of kidney injury in NOD mice has been studied by Riera et al., who demonstrated the development of mild diabetic kidney disease lesions namely albuminuria, matrix mesangial expansion, and podocyte loss at 40 days after diabetes onset [20].

Among the type 2 classical models of diabetes, different options, such as genetic manipulations, spontaneous genetic alterations, or environmentally induced obesity, have been used. High fat diet feeding is a method that induces impaired glucose tolerance and type 2 diabetes [21,22,23]. Spontaneous or genetically-induced obese rodents are typically used as type 2 diabetes models. New Zealand Obese (NZO/H1Lt) model is an inbred polygenic mouse model that develops obesity and type 2 diabetes. Adiposity in the NZO mouse is driven by hyperphagia, which might be ascribed to leptin resistance, as these mice are hyperleptinemic at 9–12 weeks of age [24]. Leptin (*ob/ob* mice) [25] or leptin receptor (*db/db* mice or Zucker diabetic fatty rats) [26,27,28] deficient rodent models are also used to study DN. These rodent models developed type 2 diabetes secondary to increased food intake, because they have an absence of satiety-related sensation. They manifest hyperphagia, obesity, insulin resistance, and hyperglycaemia. A high fat diet might also be administered to other diabetic experimental models, which *per se* might promote renal injury, although the animals do not exhibit the features of human DN [11].

Although these classical models simulate a diabetic condition, most of them do not show all of the DN features mentioned before. DN has a multigenic and environmental origin and it is difficult to identify and manipulate all the factors that contribute to this condition. Therefore, efforts have been done to improve the existing models or create new ones to study the DN pathology. Different approaches have been employed, including backcrossing to a diabetic susceptible background and genetic modifications (knockout and transgenic models). These diabetic models offer a wide and improved range of disease severity, but to date, none of them accomplishes all of the features of human DN.

### 2.1. Diabetes Susceptible Background and Strain Crossing Models

The classical diabetic models can be crossed back to a diabetes susceptible genetic background, such as BTBR [29], C57BL/6 [30], or DBA/2 [31], among others (Table 2) [13,32]. One of the most used mouse strain is the black and tan Brachyuric (BTBR) *ob/ob* (leptin deficient) mice crossed with C57BL/6 mice that was characterized by Clee et al. [33]. This is a robust and progressive model of DN that develops severe type 2 diabetes, insulin resistance, and progressive renal damage. These mice show proteinuria after four weeks of age, hypertrophy and accumulation of mesangial matrix after eight weeks, glomerular lesions after 20 weeks, and increase in glomerular basement membrane (GBM) thickness after 22 weeks. Focal arteriolar hyalinosis, diffuse mesangial sclerosis, mesangiolysis, mild focal interstitial fibrosis, and loss of podocytes can also be observed [29]. Strain crossbreeding can also be performed to better mimic DN, such as the inbred congenic strain NONcNZO10/LtJ, a model of polygenic type 2 diabetes derived from a cross between the non-obese non-diabetic (NON/LtJ) and the New Zealand Obese (NZO/H1Lt) murine strains [34,35]. NON/LtJ strain provides to NZO/H1Lt a more severe progressive development of glomerulosclerosis. However, this model develops atypical lesions of DN, such as acute interstitial nephritis, intraglomerular capillary thrombi, and lipid deposition [34,35].

### 2.2. Knockout Diabetic Models

The deletion of genes involved in cardiovascular and renal protection is also usually used to increase the DN features of the classical diabetic nephropathy rodent models. Examples of these type of models are *db/db* eNOS^−/−^ mice, the bradykinin 2 receptor (B2R) deficient, or the decorin deficient mice (Table 2).

Endothelial nitric oxide synthase (eNOS) deficiency in *db/db* mice is an improved model of DN, as the inhibition of nitric oxide formation causes hypertension and endothelial disfunction, which increases the diabetic kidney lesions when compared to *db/db* [10,50]. eNOS deficient mice have been developed in C57BL/6 and C57BLKS backgrounds. C56BL/KsJ *db/db* eNOS^−/−^ mice develop albuminuria, hypertension, mesangial matrix expansion with nodules, mesangiolysis, increased GBM, arteriolar hyalinosis, and moderate tubulointerstitial fibrosis [50,59]. The reduction of the glomerular filtration rate (GFR) is observed at 26 weeks of age [50]. Another knockout model of DN is the *Ins2^Akita/+^ Bradykinin 2 receptor (B2R) null* that results from the combination of crossing Akita mice that has a single point mutation in the insulin 2 gene (Ins2C96Y) -which results in an amino acid change- and a mice strain with a knockout mutation in the gene coding for the bradykinin 2 receptor (B2R) [51]. Bradykinin is an endogenous angiotensin converting enzyme (ACE) inhibitor that acts via the Bradykinin 1 (B1R) and 2 receptors (B2R). B1R is mainly expressed during tissue damage and it is associated to noxious effects. In contrast, B2R is constitutively expressed and its bradykinin-induced activation has cardiovascular and renal beneficial effects [60]. Therefore, B2R deficiency animals show cardiovascular and renal complications [61,62]. In particular, *Ins2^Akita/+^ Bradykinin 2 receptor (B2R) null* mice develop albuminuria, mesangial expansion, mitochondrial DNA damage (senescence) in the kidneys, and other tissues after six months. However, no changes in glomerular endothelial cells or podocytes were observed [51,52]. Decorin deficiency is also associated to accelerated kidney damage, as this protein is involved in inflammatory and fibrosis processes by binding growth factors such as transforming growth factor β (TGFβ), platelet derived growth factor (PDGF), and epidermal growth factor (EGF) [63,64,65]. Low-dose STZ type 1 diabetes induction in Decorin null C57BL/6J mice show advanced DN as the TGFβ-mediated processes, such as fibrosis and inflammation, are constantly stimulated in this model. When compared to wild-type diabetic mice, these animals show increased urine albumin excretion and a decrease in renal function. They also develop an increase in mesangial matrix expansion and kidney inflammation (macrophage infiltration and up-regulation of Nox4). Nodular sclerosis or tubulointerstitial lesions are not observed [53].

### 2.3. Transgenic Diabetic Models

The overexpression of genes related to RAS or the glucose metabolism is also a usual approach to worsening DN lesions in diabetic mouse models. The overexpression of human renin in mouse models (TTRhRen mice) produces hypertension secondary to a chronic activation of the systemic RAS [54] and, therefore these animals are more prone to developing DN when induced by STZ [8,55]. A diabetic phenotype can also be achieved by crossing the TTRhRen mice with OVE26 diabetic mice, that are deficient in insulin production for calmodulin overexpression in the pancreatic β cells (OVE26-TTrhRen double transgenic). This mouse model develops significant albuminuria, mesangial expansion, tubulointerstitial fibrosis, and a decline in renal function at 20 weeks [54]. Hyperreninemic rat models are also available, such as the CYP1a1mRen2, which express the murine renin–2 gene (mRen2) under the Cytochrome P4501a1 promoter. This promoter allows the control of the timing and severity of hypertension by adjusting the concentration of indole-3-carbinol in the diet [56]. The hypertensive CYP1a1mRen2 rats after STZ-diabetes induction develop marked albuminuria (500–fold), glomerulosclerosis, and tubulointerstitial fibrosis. However, this model lacks in some of the classical features of DN, such as arteriolar hyalinosis [55,56]. Furthermore, the overexpression of some genes related to glucose metabolism has also been used to increase the severity of the diabetic phenotype and, consequently, the DN features. Examples of this approach are the GIPR^dn^ and the GLUT-1 transgenic mice, which exhibit renal changes that closely resemble diabetes-associated kidney alterations. GIPR^dn^ transgenic mice express a dominant-negative mutation of the glucose-dependent insulinotropic polypeptide (GIP) receptor in the pancreatic β-cells. These mice show an incorrect development of the pancreatic islets, producing an impairment of insulin secretion. Consequently, these animals present an early diabetes onset without any other metabolic alteration and progressive kidney disfunction [57]. Interestingly, C57BL/6 mice locally overexpressing GLUT-1 in the glomeruli do not show hyperglycaemia, even though they develop albuminuria, renal function decline, and kidney morphological changes that are similar to those observed in human DN. This model shows a slower kidney disease progression when compared to C57BL/6 *db/db* animals [58].

## 3. Genetic Background

In humans, several lines of evidence support an inherited genetic predisposition to DN; hence, only a subset of individuals with type 1 or type 2 diabetes will develop this disease [66]. The cumulative incidence of diabetic retinopathy increases almost linearly with the duration of diabetes, whereas DN occurs in approximately 40% of individuals with long-standing diabetes mellitus (DM) [67]. Familial clustering and ethnic variation are associated to the susceptibility to suffer DN in both type 1 and type 2 Diabetes [68,69]. Genetic studies conducted in patients with DN have focused in finding genes with biological roles in the pathogenesis of this disease. Many genes and single-nucleotide polymorphisms (SNPs) have been reported to be significantly associated with DN, as in most multifactorial diseases [18]. Genome-wide association studies (GWAS) have had a crucial role in identifying SNPs related to common complex diseases that are associated with kidney phenotypes. To date, although several large GWAS studies have been performed, the progress identifying susceptibility genes for DN has been slow [18], possibly related to the imprecise phenotyping and the existence of multiple genes with small effects influencing in DN development [70].

Similarly to humans, genetic factors have an important role in determining the susceptibility to DN lesions in rodent models. In some studies, strong effects of genetic background on the severity of kidney disease across different experimental platforms have been documented, including STZ, Akita, and db/db models [71,72,73,74]. STZ induced diabetic mouse has been one of the most used animal model to study early diabetic nephropathy (DN), mainly related to their low cost, and the easy experimental procedure. However, the power to induce diabetes with STZ is highly dependent on the mouse strain —DBA/2 > C57BL/6 > MRL/MP > 129/SvEv > BALB/c— and the gender. Male mice are more susceptible to STZ-induced diabetes, and develop more profound renal injury as compared to their female counterparts. The albuminuria levels were greatest in the DBA/2 and KK/HIJ strains, which also had the most marked hyperglycemia [9,19]. Regarding the Akita diabetic model, the DBA/2 and 129SvEv Akita mouse strains are more susceptible to diabetic kidney injury when compared to the C57BL/6 Akita mice. C57BL/6 strain with Akita mutation develops a modest increase in albuminuria, mesangial matrix, and basement membrane thickening [32]. Models of type 2 diabetes typically use genetically obese rodents, such as leptin deficient mice (*ob/ob* mice) or inactivating mutations in the leptin receptor (*db/db* mice or Zucker rats), as mentioned before. *db/db* mice on the C57BLKS background results from the modification of C57BL/6 to contain part of the DBA/2J strain and it develops several features of DN that are mild under the C57BL/6 background. *ob/ob* mice in the C57BLKS background exhibit severe hyperglycaemia, whereas *ob/ob* mice in the C57BL/6J background develop only mild hyperglycaemia, and hyperplasia of the pancreatic ducts. Interestingly, the C57BLKS mouse is more susceptible to the effect of the β-cell toxin STZ as compared with the C57BL/6 strain [75].

## 4. Assessment of Renal Function in Experimental Models

Following the characteristics that were established by de AMDCC, DN animal models are defined by two major clinical findings: a decrease in GFR and an increase of albumin urinary excretion (Table 1). However, not all models develop both features, and a significant variability between them exists [9]. For these reasons, GFR and albuminuria assessment are crucial for phenotyping and understanding every DN model. In the last years, several methods for GFR measurement and urinary albumin quantification have been developed.

### 4.1. Glomerular Filtration Rate Assessment

GFR is considered to be the best parameter for the evaluation of renal function. Presently, several methods have been developed for its assessment in animal models. GFR is usually estimated by measuring the clearance of an endogenous or an exogenous substance. Ideally, this substance should be filtered in the glomerulus and excreted in urine, without suffering any tubular reabsorption or secretion that interferes with the measurement. Moreover, the intrinsic generation variability of the endogenous markers or the extrarenal elimination also affects correct GFR estimation. Creatinine, which is an endogenous compound commonly used to assess GRF humans, is not accurate in humans and mice, because it is also secreted to urine by proximal tubular cells. In some mice strains it is described that tubular secretion of creatinine might account for up to 50% of creatinine clearance [13]. Therefore, renal creatinine clearance exceeds GFR and it would overestimate the latter. Inulin is the classically employed tracer for GFR measurement. This exogenous compound is filtered in the glomerulus and is completely excreted in urine. Currently, inulin is usually used bound to fluorescein isothiocyanate (FITC-inulin) for facilitating its measurement in plasma and urine samples. Other tracers that are based on inulin, such as radioactive Carboxyl-^14^C-inulin, are less commonly used. Recently sinistrin—an inulin-like molecule—is replacing inulin at the experimental level. This substance is also attached to FITC, with the advantage that dialysis for eliminating the unbound FITC molecules is not required before the experiment [76]. Another exogenous marker frequently used for GFR assessment is iohexol, an iodine-based radiographic contrast agent with characteristics that are similar to inulin. Tubular reabsorption has been described for this marker, and iohexol underestimates GFR when compared to inulin [77]. Moreover, the iodine that is part of its structure can cause tubular toxicity when employed at high doses [78]. Other markers, such as ethylenediaminetetraacetic acid (EDTA) or diethylenetriaminepentaacetic acid (DTPA) bound to Technetium-99m, have also been described for GFR assessment.

#### 4.1.1. Creatinine Clearance

The creatinine clearance method measures creatinine from a timed urine collection (usually a 24-hour urine collection) and serum creatinine concentration during the collection period [79]. Measurements are usually performed by the high performance liquid chromatography (HPLC) method, as it is more accurate than the alternative Jaffe alkaline picrate method, which overestimates creatinine concentration [13]. The GFR measuring method that is based on creatinine clearance, which is commonly used in clinical practice, has its limitations as it overestimates GFR. Moreover, creatinine serum values can be within normal range, even with more than a 50% decrease in GFR [80]. For these reasons, and although the quantification of creatinine is an easy and affordable technique, creatinine clearance measured with HPLC is not an accurate method for estimating GFR [13].

#### 4.1.2. Anesthetized Inulin Clearance

Anesthetized inulin clearance is a method that is based on radioactive tracer injection. It is a complex and invasive technique that requires left carotid artery and left jugular vein cannulation for continuous intravenous infusion. Bladder cannulation is also necessary for facilitating urine sample extraction. Exogenous Carboxyl-^14^C-inulin is infused at specific rate until it reaches steady state. Renal function is then measured by the quantification of Carboxyl-^14^C-inulin in plasma and urine [12]. The obtained values accurately measure GFR and are usually used as reference to compare the values obtained with other measurement methods. However, the technique is so invasive that it is not commonly used in experiments, and it does not allow multiple measurements over long periods in the same experiment.

#### 4.1.3. Steady State Inulin Clearance

This method is a less invasive approach for measuring steady state inulin clearance in conscious mice. It is characterized by the intraperitoneal implantation of an exogenous fluorescent tracer (FITC–Inulin) mixed with an osmotic solvent in a mini pump [12]. When FITC-Inulin reaches steady state in plasma, short and serial determinations of GFR could be performed in no anesthetized mice [12].

#### 4.1.4. Non-Steady State Inulin Clearance

It is another technique that is based in inulin clearance. The decay rate of FITC-inulin is measured after an intravenous bolus injection, instead of calculating GFR while using steady-state of this tracer. This method is less invasive than the previous one and allows performing repeated measurements over longer periods. After a bolus injection, plasma samples are obtained at regular intervals from venous puncture and used for tracer measurement. The results over time allow for researchers fitting a curve that represents FITC-inulin decay rate. Two components of this curve are usually recognized; the first component represents the redistribution of inulin from plasma into the extracellular space and the second reflects the renal clearance of inulin.

#### 4.1.5. Transcutaneous Sinistrin Clearance Measurement

**Figure 1:**
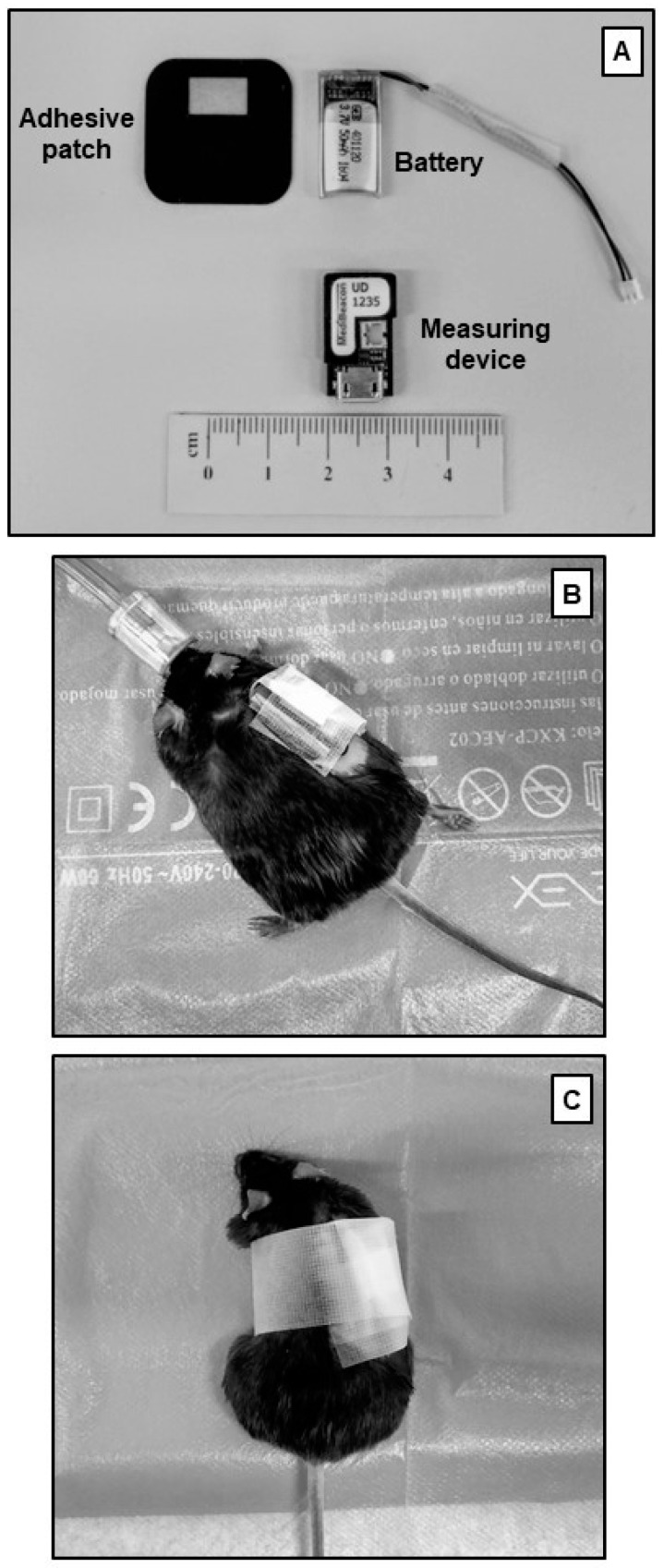
**Transcutaneous glomerular filtration rate measurement technique.** (**A**) Transcutaneous measuring device and its components. (**B**), The device is placed in the shaved back of the mouse anesthetized with isoflurane. Adhesive tape is also used to properly fix the device and avoid movement artefacts. (**C**), Once attached and after fluorescein isothiocyanate (FITC)-sinistrin administration, the mouse can move freely during measuring period.

**Figure 2:**
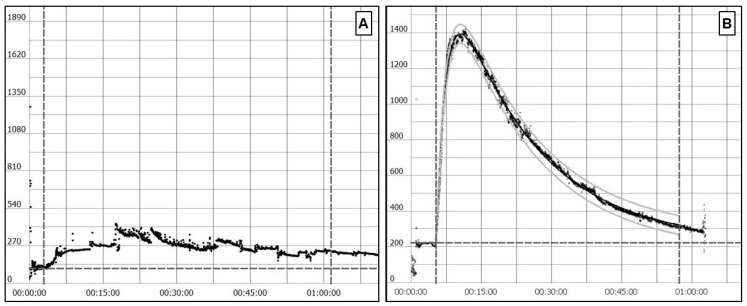
**Transcutaneous measuring results fitted to a decay curve.** (**A**), Wrong transcutaneous glomerular filtration rate (GFR) measurement. After skin background measurement there is no signal peak, as FITC-sinistrin was not correctly administered intravenously. (**B**) Correct transcutaneous GFR measurement. The curve shows a basal measurement of the skin background followed by a signal peak after FITC-sinistrin intravenous bolus administration.

Transcutaneous sinistrin clearance is an even less invasive method that can be performed in conscious mice. It does not require the tracer to reach steady-state and measurement is also done after an intravenous bolus injection. In contrast to the non-steady state inulin clearance, with this method there is no need to obtain plasma samples. A miniaturized device is placed in the shaved back of the mouse while it is anaesthetized. This device allows for a continuous transcutaneous measurement of the decay rate of the fluorescent agent attached to sinistrin (Figure 1). Usually, isoflurane—a fast-acting inhaled anesthetic—is used for anesthesia, because the device placement and bolus injection are short procedures. Once the device is placed and the tracer agent administered, the mouse can freely move during the measuring time. The obtained results allow for fitting a curve that estimates the half-life of FITC-sinistrin, which can then be converted to GFR by a validated formula [76] (Figure 2).

#### 4.1.6. Iohexol Clearance

Iohexol is an iodinated, non-ionic radiographic contrast agent that has been proposed as an alternative exogenous marker for GFR evaluation [77]. Recent studies in rats from Carrera et al. [81] showed an easy iohexol clearance measurement method, which prevents urine sampling, anesthesia, and animal catheterization. This procedure is similar to the non-steady state inulin clearance method. First, a blank blood sample is obtained. Next, an iohexol bolus is injected to the animal and blood samples are drawn at fixed times after the administration for plasma clearance evaluation. Plasma iohexol concentrations are then measured by HPLC [81]. The tracer concentration decay is finally analyzed according to a two-compartment kinetic model.

In summary, there are multiple techniques for GFR measurement. The most widespread is non-steady state inulin clearance, as it is a barely invasive method that allows multiple measurements over time in the same mice. Therefore, it is possible to measure GFR before and after administering experimental treatments, and without causing any significant harm to the animal that might introduce biases into the procedure. However, other new methods, such as the transcutaneous sinistrin clearance measurement, are less invasive and easier to perform than non-steady state inulin clearance. For this reason, we think that the transcutaneous method will be widely expanded in the future.

### 4.2. Albuminuria Measurement

Albuminuria is the other clinical variable that should be measured and correlates with diabetic renal lesion. As glomerular filtration barrier is damaged in DN, its selectivity is lost, and bigger proteins, such as albumin, are filtered and excreted in urine. Thus, the study of kidney function goes commonly through the identification of albuminuria in experimental animals. For this purpose, it is necessary to obtain the mouse urine and several techniques have been described. One of the most widespread procedures is the use of metabolic cages that allow for obtaining an isolated urine sample or even a 24-hour urine collection, avoiding its contamination by faeces. The problem with the latter is that metabolic cages are not always available in every laboratory, so alternative options for urine collection have to be considered. Other techniques, such as abdominal massage while holding the mouse or placing it over a 96-well plate for some time [9], will help to obtain the urine that can then be aspirated while using a pipette without contamination by faeces.

The quantification of urinary albumin concentration is the most significant analysis for the evaluation of diabetic kidney disease, as it is the predominant urinary protein in DN [82]. Two main methods can be employed for albuminuria assessment: immunochemical techniques and size-exclusion HPLC methods [82]. Urine albumin can be measured in the spot urine or in a timed urine collection (24-hour urine collection). In clinical practice, an isolated morning urine sample is habitually employed, as it is easier to collect. Urine albumin is normalized by creatinine, namely the urine albumin-to-creatinine ratio (UACR). The UACR is calculated dividing albumin concentration by creatinine concentration, and the ratio is expressed in mg/g or μg/mg. In humans, when total 24-hour proteinuria is lower than 3 grams, UACR correlates well with total 24-hour albumin excretion and UACR value can replace the latter. In mice, albuminuria in 24-hour urine and UACR in spot urine samples are not always concordant. Strain dependent differences have been described that could be related to variations in creatinine excretion between them. For these reasons, AMDCC investigators recommend reporting both measurements [13].

UACR calculation requires albumin and creatinine measurement in urine. Both of the values can be measured while using an indirect competitive ELISA (Albuwell M) and the Creatinine Companion kit (Exocell) [20]. The Albuwell M ELISA is a competitive immunohistochemical assay where albumin is identified by anti-albumin antibodies. This method is characterized by the existence of a solid phase of albumin in pre-coated wells and a fluid phase, corresponding to the albumin that is present in the urine sample. Therefore, the anti-albumin antibodies can react with both albumin phases; hence, the notion of competitive binding [83]. Urine albumin can be then calculated through color intensity, which is inversely proportional to the logarithm of albumin in the fluid phase. The creatinine companion kit was created to be used in combination with the ELISA albumin-detection kit explained above. This kit is based in an adaptation of the alkaline picrate method firstly described by Jaffe. The procedure includes absorbance determination in each sample before and after the addition of acid, which is added to the solution in order to correct the color generation, due to the presence of substances that interfere in the optical density [84].

In daily practice, UACR is an easy and accessible method to perform that does not require metabolic cages. However, UACR values are not comparable to 24-hour albuminuria and should be analyzed as different variables when evaluating an experimental procedure. Currently, UACR is an accepted method for the diagnosis and management of DKD in both human and experimental animal models.

## 5. Renal Lesions and Morphometry in Experimental Models of DN

The AMDCC established the histological findings that validate an ideal DN animal model (Table 1). Most classical DN rodent models, such as NOD, Akita, or db/db mice, only exhibit incipient diabetic nephropathy changes. Mild mesangial expansion, glomerular hypertrophy, and GBM thickening are the usual histological findings (Table 1). These lesions are equal to classes I and IIa of the human DN classification [85]. Genetic manipulation and the use of susceptible strains has led to the development of more robust rodent models that develop established DN lesions, such as severe mesangial expansion, nodular sclerosis, or even glomerulosclerosis. Renal morphological studies are useful for assessing the severity of the employed diabetic model and the effect of experimental procedures on DN. Here, we present the principal methods used to assess histopathological lesions.

### 5.1. Mesangial Matrix Expansion Measurement

Mesangial matrix expansion is one of the most important features to validate an established DN model [75]. In vivo and in vitro studies provide evidence that hyperglycemia activates various pathways that lead to increased synthesis of extracellular matrix components and mesangial expansion, with the latter being a histological sign of DN [86,87]. The evaluation of extracellular matrix in glomeruli is usually performed by the Periodic Acid Schiff (PAS) reaction, which is an easy to perform and economical stain [88]. The reactivity of the PAS staining is based on the interaction with monosaccharide units. In consequence, the extracellular matrix (ECM)), which is rich in glycosaminoglycans is highlighted. The limitations of this staining rely on the difficulty to discriminate between extracellular matrix and cytoplasm, because it reacts with the carbohydrates of both compartments [13]. The assessment of mesangial matrix expansion and mesangiolysis can also be performed by the silver methenamine reaction, a more specific extracellular matrix staining. However, it requires a more complex staining process and reagents are more contaminating [13]. The silver reaction provides a clear distinction between glomerular capillary loops and mesangial matrix, allowing for better quantification of the glomerular matrix.

Another challenge of mesangial matrix and kidney histology evaluation is that data are usually classified categorically. Moreover, trained professionals are required to evaluate the samples and an interindividual variability is frequent when measuring pathological lesions. Categorical data are also difficult to handle statistically and there is an increased risk of bias [89]. Semi-quantitative methods were first developed to overcome these problems. The results are adjusted to the same scale using validated scores for each element of the kidney histology. However, semi-quantitative techniques have a lower sensitivity, do not offer an accurate quantification, and are also subjected to scoring bias [90]. For these reasons, they have been progressively replaced by digital quantitative methods at an experimental level. Automation and computer advances in the last two decades made the use of digital imaging and the development image analysis software accessible. Nowadays, several image analysis programs are available for these tasks, but probably one of the most widespread is the open license Image J software [90].

PAS stained slides can be digitalized while using a camera adapted to light microscopy. Subsequently, using the image J analysis software, the glomeruli of each slide can be automatically or manually identified. For mesangial matrix evaluation, manual identification of glomerular area is usually performed with this program. A correct measurement requires at least 20 digitalized glomeruli at hiliar cross-sections to allow for whole glomerular tuft observation. In light microscopy, glomeruli are usually evaluated and digitalized at ×400 magnification. Afterwards, the glomerular area is manually outlined using the mouse. The outlined area is named by Image J as region of interest (ROI). Once the ROI of the 20 glomeruli are identified, we can obtain a mean value of the glomerular area in that sample. Moreover, the degree of the mesangial matrix expansion can be evaluated by measuring the PAS-positive material within the mesangial region and then adjusting it to the whole glomerular tuft area [20,91]. Nevertheless, the manual identification of kidney structures needs trained professionals for adequate measurements and are susceptible to observer bias. To minimize this bias, it is recommended that two or more different observers evaluate the samples. In addition, automatic glomerular identification and kidney structure segmentation programs have also been developed, which facilitate renal histology analysis and make it accessible to less trained researchers [89].

### 5.2. Podocyte Number and Density

The reduction of podocyte number and density antedates glomerulosclerosis and it is associated with progression to ESRD [92]. Therefore, podocyte counting is another feature that assesses glomerular damage in DN. Wilms Tumor 1 (WT1) is a transcription factor that is highly expressed in these cells nuclei [92], thus the quantification of the number of podocytes is normally performed by WT1 staining. The detection of WT1 by immunochemistry or immunofluorescence allows for estimating podocyte density with a single histologic section, rather than other techniques, such as the dissector/fractionator approach [92], which are more complex. Primary WT1 antibody, in contrast with DAPI stain, which labels all cell nuclei, brings a useful immunofluorescence assay to analyse podocyte loss. However, simple podocyte nuclei counting may overestimate the number of podocytes by 200–300% due to the fact that podocyte nuclei are larger in relation to sample section thickness [92]. WT1 immunostaining is performed in deparaffined and rehydrated murine kidney sections. The sections are heated in a specific buffer for antigen retrieval and are then incubated with anti-WT1 primary antibodies. The produced antigen-antibody complex is highlighted while using horseradish peroxidase conjugated to a secondary antibody through avidin-biotin complexes, and stained with diaminobenzidine (DAB) substrate. Sections are usually counterstained with hematoxylin, as it outlines cell nuclei and facilitates podocyte count. Finally, each section is examined by light microscope (LM), photographed with a digital camera, and analyzed by an image-analyzer program, like Metamorph Imaging System or Image J [20,93]. The total podocyte value is then divided by the total cell nuclei stained with hematoxylin in each glomerular profile to obtain the podocyte number per glomerulus.

Sanden et al. described another method for podocyte (P) count based in thin/thick sample correction [94]. The authors support the podocyte measurement adjusted to glomerular volume (GV/P), and define another value, known as podocyte density. This alternative system is supported by the fact that differences in podocyte number between thin and thick sections are directly proportional in the differences in sample thickness [94]. The mean values of P number are obtained following the same procedure explained above for thin and thick slices tissues (for example 3 and 9 μm), but without previous glomerular profile selection. Besides, glomerular tuft area (GA) is measured using an image-analyzer system. Once all of these data have been collected, the results can be expressed as mean podocyte number per glomerular tuft area (P/GA). The difference in podocyte number between thin and thick sections is directly proportional to the previously known difference between section thickness (P/GA^thick^ − P/GA^thin^ = P/GAΔ), as mentioned before. Therefore, we can obtain the GV/P value by dividing the known difference thickness between the samples (6μm) by the P/GAΔ value [94]. Hereby, podocyte density overestimation is prevented by nulling the possible bias of error, such as changes in podocyte nuclear size, loss of tissue, or double counting [94].

### 5.3. Glomerular Basement Membrane Thickness Measurement by Electron Microscopy

Another morphometric analysis is the examination of glomerular abnormalities by transmission electron microscope (TEM), which allows for high-resolution visualization of ultrastructure abnormalities in the kidney. Using TEM, lesions seen in DN models, such as GBM thickening, can be evaluated. In fact, the evaluation of the GBM thickness using TEM permits determining the presence of dense material in the glomerular compartment. TEM should be performed with tissues with at least one glomerulus and it requires an image analysis software and a ponderation system for quantification [95]. For ultrastructural morphometric measurements, biopsy derived kidney tissue should be cut in 1 mm^3^ fragments and then fixed in 1 to 4% glutaraldehyde in sodium cacodylatebuffer. A post-fixed treatment with 2% osmium tetraoxide in sodium cacodylatebuffer should be also performed, followed by dehydration in ethanol and embedment in Epon [20,96,97]. Subsequently, thin sections (usually of 90nm) are stained with uranyl and lead. Sections are then photographed at low and high magnification while using ultrahigh resolution TEM equipped with a digital camera. High magnification micrographs are useful for determining GBM thicknesses, whereas low magnification images are normally used for mesangial evaluation by MacLeod et al.’s approach [98,99] as an alternative of the PAS-positive method explained above.

GBM width can be measured with the orthogonal intercept method from Jensen et al. [100], based on the fact that, only when the sectioned samples have a uniform orientation distribution, it is possible to apply the correction factor to avoid the overestimation of membrane thickness [96,100]. Samples should be placed at a random distribution where each possible angle is equally likely to occur to obtain uniform orientation. This is achieved by transferring the samples to capsules with plastic or resin without knowing the GBM distribution [96]. Once this is performed, the resulting electron micrographs are placed under a grid. At all places where a grid line intercepts an endothelial-GBM interface, an orthogonal line is drawn and its length is determined [20]. The arithmetic mean of these orthogonal intercept lengths, multiplied by π/4, provide the measurement of the GBM thickness width.

### 5.4. Tubular Injury

Tubular damage also plays an important role in DN progression. These lesions are accompanied by abnormal accumulation of extracellular matrix in the interstititum, leading to interstitial fibrosis. Interstitial fibrosis and tubular atrophy (IFTA) are frequent in human DN. However, in experimental models of diabetes it is uncommon and it has mainly been described in more recent ones. Image analysis techniques are employed for tubular injury assessment. Specific stains, such as the Masson trichrome, Picrosirius red stain, or collagen immunohistochemistry methods, can be employed as markers for fibrosis measurement. Afterwards, with the use of image analysis software, the color threshold is set, and the stained area is measured within the previously established ROI [90]. Another technique to evaluate tubular cell injury is the measurement of the tubule cross-sectional diameter and the tubular cell height. Tubular cell height is measured from the basal surface to the apical membrane [101]. Damaged tubular epithelial cells lose their cuboid shape and height. An adequate number of tubules should be measured (50 randomly selected tubules in 10 non-overlapping fields) for avoiding biases.

## 6. Conclusions

Currently there is a wide variety of diabetic animal models that resemble pathophysiological mechanisms of both type 1 and 2 diabetes. In the last decades, important advances have been made in creating mouse strains that develop clinical and histological findings mimicking overt DN. Many of these models are obtained through the conjunction of mutations that contribute to diabetes development itself, and modifications of cardiovascular regulatory pathways. In this line, diabetic mice with altered nitric oxide synthase, bradykinin receptor loss, or renin overproduction can be found for experimental use. Researchers should be aware about the model they choose, as not all physiological pathways can be studied in each model. In addition, there are several techniques that the researchers that are involved in the study of experimental models of renal diseases should know to assess renal function and histological lesions, which are key to understanding the studied experimental models and the effects of the new therapeutic tools.

## Figures and Tables

**Table 1:** Characteristics of acceptable animal models with diabetic kidney disease established by the Animal Models of Diabetic Complications Consortium (AMDCC). Adapted from AMDCC [12].

Clinical features
-Greater than 50% decline in GFR over the lifetime of the animal. -Greater than 10-fold increase in albuminuria compared with not affected controls for that strain at the same age and gender.
**Pathological features**
-Mesangial matrix expansion. More severe models may show nodular sclerosis. -Any degree of arteriolar hyalinosis. -Glomerular basement membrane thickening by more than 50% over baseline. -Tubulointerstitial fibrosis (usually seen in severe DN models).

**Table 2:** Experimental models of diabetic nephropathy (DN).

Type	Name	Strain	Diabetic Model	Diabetes Type	Systemic Metabolic Features	Albuminuria Increase (Age/Grade)	eGFR 50% Decline (Age) *	Kidney Features	References
**Induced**	Streptozotocin (STZ)	Any (mice/rats)	STZ-induced	T1DM	Hyperglycaemia in 90% of the STZ-induced animals. In mice, C57BL/6 and DBA/2 are high responders to STZ.	**Mice:** - BALB/c, DBA/2 and KK/HIJ strains: at 16 weeks of DM onset/10-fold - C57BL/6, MRL/Mp, 129/SvEv and A/J strains: minor or no changes at 16 weeks.	No decline of eGFR at 15 weeks of DM onset in C57BL/6J, DBA/2J, FVB/NJ, MRL/MpJ, A/J, and KK/HlJ strains (Hyperfiltration, except for A/J strain)	Mesangial expansion (more important in DBA/2 and KK/HIJ strain)	Zhonghua et al. 2005 [9] Gurley et al. 2006 [19] Deeds et al. 2011 [36]
						**Rats** -Wistar-Furth: 1 week post-STZ/10-fold and 4 weeks post-STZ/20-fold - Sprague-Dawley: 6 weeks post-STZ/400-fold	Wistar-Furth: no eGFR decline at 4 weeks after STZ (hyperfiltration).	Sprague-Dawley: mild mesangial expansion. Increase of fibronectin expression.	Palm et al. 2004 [37] Danda et al. 2005 [38]
	High Fat Diet (HFD)	Any (mice/rats)	Diet-induced	T2DM	Obesity, dyslipidemia, hypertension, hyperglycemia. Increases diabetic features and DN severity in most models.	**Mice** - C57BL/6 at 32 weeks/1.5 fold - db/db at 16 weeks (2 weeks of HFD)/3-fold. (vs. same model on chow diet).	No decline after 22 weeks (early hyperfiltration).	C56BL/6: 2 to 4 weeks of HFD: mesangial matrix expansion. 8 weeks of HFD: increased inflammation. 16 weeks of HFD: glomerular fibrosis.	Wei et al. 2004 [39] Zhang et al. 2012 [40] Glastras et al. 2016 [41] Xu et al. 2017 [42]
						**Rats** Sprague-Dawley: after 4 weeks of HFD/4-fold (vs. Chow diet)	Sprague-Dawley: No decline after 14 weeks of DM induction.	Sprague-Dawley: GBM thickening and mesangial matrix expansion. Not tubular atrophy.	Dong et al. 2019 [43] Danda et al. 2005 [38]
	STZ + HFD	Any (mice/rats)	Diet and STZ-induced	T2DM	Add-on effects vs. STZ or HFD models	**Mice:** -C57BL/6J: DM induction with 7 weeks of HFD + single dose STZ. After 15 weeks/3-fold (vs. Chow diet). -C57BL/6J: DM induction with 5 weeks of HFD + single dose STZ. After 24 weeks/2-fold (vs. Chow diet) and mild increase (vs. HFD).	C57BL/6J: DM induction with 5 weeks of HFD + single dose STZ. After 24 weeks/Mild increase in serum creatinine levels (vs. chow diet but not vs. HFD).	C57BL/6J: mesangial expansion and tubular vacuolization. Lipid deposition.	Kim et al. 2016 [44] Glastras et al. 2016 [41]
						**Rats** Sprague-Dawley: DM induction with 5 weeks of HFD + single dose STZ. After 6 weeks/400-fold	Sprague-Dawley: No decline after 14 weeks of DM induction.	Sprague-Dawley: Increased mesangial matrix expansion. Increased fibronectin and collagen expression. (vs. STZ and HFD)	Danda et al. 2005 [38]
**Spontaneous mutations**	Akita mice	DBA/2, 129/SvEv, C57BL6	Akita	T1DM	**Hyperglycaemia**, hypoinsulinemia, polydipsia, and polyuria at 3–4 weeks of age	Depending on the strain, at 6 months of age/8-fold (DBA/2 x C57BL/6), 2-fold (DBA/2 and 129/SvEv). No change in C57BL/6.	No decline at 6 months of age (hyperfiltration)	Mesangial matrix expansion	Gurley et al. 2010 [45]
	Non-obese diabetic (NOD) mice		NOD	T1DM	Autoimmune diabetes onset at 12–13 weeks of age hyperglycaemia	21 days of diabetes onset/10-fold 40 days of diabetes onset/20-fold (vs. NOR mice)	No decline after 40 days of diabetes onset (hyperfiltration)	Early kidney disease	Riera et al. 2014 [20]
	ob/ob mice (leptin deficient)	C57BL/6	ob/ob	T2DM	Obesity, hyperglycaemia, insulin resistance	22 weeks/4-fold (vs. WT)	-	Mesangial matrix expansion	Hudkins et al. 2010 [29]
	db/db mice (leptin receptor deficient)	C57BL/6 C57BLKS	db/db	T2DM	Hyperglycaemia (C57BLKS more susceptible), insulin resistance, hypertension, hyperphagia, obesity	18 weeks/6-fold (vs. db/m)	28 weeks (in aprox. 40% of the cases, vs. db/m)	Mesangial matrix expansion	Bivona et al. 2011 [46]
	Zucker diabetic Rats (leptin receptor deficient)	Merck M crossed with Sherman rats		T2DM	Hyperphagic and hyperinsulinemic, hyperglycaemia, hyperlipidemia	16 weeks/200-fold 26 weeks/1000-fold (vs. Lean)	No decline after 26 weeks (hyperfiltration)	Glomerular and tubular damage	Hempe et al. 2012 [28]
**Polygenic**	Non-obese Non-diabetic (NON) mice		NON	T2DM	Impaired glucose tolerance (in 50% of the cases)	UK	Increased serum creatinine at 6 months	Glomerular lesions not resembling DN	Watanabe et al. 1991 [35]
	New Zeland Obese (NZO)/HlLt mice		NZO/H1Lt	T2DM	Obesity and 50% are diabetic (hyperinsulinemia, insulin resistance, glucose intolerance)	UK	UK	UK	Haskell et al. 2002 [24]
**Backcrossing models**	BTBR-ob/ob mice	BTBR crossed with C57BL/6	ob/ob	T2DM	Insulin resistance, hyperglycaemia, hypertension, hyperphagia, obesity	8 weeks/2-fold and 20 weeks/10-fold (vs. BTBR WT)	No decline at 24 weeks (hyperfiltration)	Progressive renal damage, hypertrophy and accumulation of mesangial matrix (8 w), glomerular lesions (20 w).	Clee et al. 2005 [33] Hudkins et al. 2010 [29] Ericsson el al 2017 [47]
	NONcNZO10/LtJ mice	NON/LtJ + NZO/H1Lt	-	T2DM	Insulin resistance, maturity-onset hyperglycaemia, visceral obesity, dyslipidemia	Albuminuria -	UK	Glomerulosclerosis, intraglomerular capillary thrombi and lipid deposition, nephritis, and Ig deposition.	Leiter et al. 2004 [34] Soler et al. 2012 [10]
**Knockout models**	eNOS deficiency mice	C57BL/6	STZ-induced	T1DM	Hypertension, hyperglycaemia	15 weeks/3-fold 30 weeks/up to 30-fold (vs. WT-STZ)	26–28 weeks (vs. WT-STZ)	Glomerular and tubulointerstitial damage.	Kanetsuna et al. 2007 [48] Nakagawa et al. 2007 [49]
	eNOS deficiency mice	C57BLKS	db/db	T2DM	Hypertension, hyperglycaemia	24–26 weeks/30-fold (vs. db/db)	24–26 weeks (vs. db/db)	Glomerular and tubulointerstitial damage	Zhao et al. 2006 [50]
	B2R deficiency mice	C57BL/6	Akita	T1DM	Hyperglycaemia, hypoinsulinemia, hypertension	6 months/4-fold (vs. Akita)	Hyperfiltration (12 weeks)	Glomerular and tubulointerstitial damage	Kakoki et al. 2004 [51] Kakoki et al. 2010 [52]
	Decorin deficiency mice	C57BL/6	STZ-induced	T1DM	Hypoinsulinemia, hyperglycaemia	6 months/2 fold (vs. Decorin +/+ STZ)	10 months (vs. Decorin +/+ STZ)	Mesangial matrix expansions and kidney inflammation	Williams et al. 2007 [53]
**Transgenic models**	TTRhRen mice	FVB/NJ	STZ-induced	T1DM	Hypertension, hypoinsulinemia, hyperglycaemia	20 weeks/3-fold (vs. WT-STZ)	18 weeks (vs. WT-STZ)	Mesangial expansion. tubulointerstitial fibrosis	Thibodeau et al. 2014 [54]
	OVE26-TTRhRen mice	FVB/NJ	Crossed with OVE26 T1DM	T1DM	Hypertension, hypoinsulinemia, hyperglycaemia	20 weeks/40-fold (vs. OVE-26)	20 weeks (vs. OVE-26)	Mesangial expansion. tubulointerstitial fibrosis	
	CYP1a1mRen2 rat (also found in literature as CYP1a1Ren2 rat)	Fisher rat	STZ-induced	T1DM	Hypertension, hypoinsulinemia, hyperglycaemia	Onset at 16 weeks. 28 weeks/10-fold (non STZ-induced vs. WT) 500-fold (STZ-induced vs. WT)	No decline at 28 weeks (vs. WT).	Glomerulosclerosis and tubulointerstitial fibrosis	Conway et al. 2012, 2014 [55,56]
	GIPR^dn^ mice	CD1	-	T1DM	Hypoinsulinemia, hyperglycaemia	20 weeks (50% of the animals) and 28 weeks (all the animals)/5-fold (vs. WT)	NA	Progressive kidney disfunction	Herbach et al. 2009 [57]
	GLUT-1 mice	C57BL/6	-	Non- diabetic	-	26 weeks/2.4-fold (vs. WT)	Unknown (25% serum creatinine increase at 26 weeks, vs. WT)	Slow kidney disease progression, increased glomerular matrix, thickened GBM and glomerulosclerosis	Wang et al. 2010 [58]

*Any technique is considered (creatinine clearance, inulin clearance, sinistrin clearance, among others). In some cases, serum creatinine increase has been indicated when eGFR was not available. eGFR: estimated glomerular filtration rate. STZ: Sreptozotocin. HFD: High-fat diet. DM: Diabetes mellitus. T1DM: Type 1 diabetes mellitus. T2DM: Type 2 diabetes mellitus. WT: wild-type. GBM: glomerular barrier membrane. UK: unknown. w: weeks.
